# Proteomic and transcriptomic analyses of early and late-chronic *Toxoplasma gondii* infection shows novel and stage specific transcripts

**DOI:** 10.1186/s12864-019-6213-0

**Published:** 2019-11-14

**Authors:** Andrew L. Garfoot, Gary M. Wilson, Joshua J. Coon, Laura J. Knoll

**Affiliations:** 1 Department of Medical Microbiology and Immunology, University of Wisconsin – Madison, Madison, WI 53706 USA; 2 Department of Chemistry, University of Wisconsin-Madison, Madison, WI 53706 USA; 3 Department of Biomolecular Chemistry, University of Wisconsin – Madison, Madison, WI 53706 USA

**Keywords:** *Toxoplasma gondii*, Cysts, Bradyzoites, RNA-seq, Proteomics

## Abstract

**Background:**

The protozoan pathogen *Toxoplasma gondii* has the unique ability to develop a chronic infection in the brain of its host by transitioning from the fast growing tachyzoite morphology to latent bradyzoite morphology. A hallmark of the bradyzoite is the development of neuronal cysts that are resilient against host immune response and current therapeutics. The bradyzoite parasites within the cyst have a carbohydrate and protein-rich wall and a slow-replication cycle, allowing them to remain hidden from the host. The intracellular, encysted lifestyle of *T. gondii* has made them recalcitrant to molecular analysis in vivo.

**Results:**

Here, we detail the results from transcriptional and proteomic analyses of bradyzoite-enriched fractions isolated from mouse brains infected with *T. gondii* over a time course of 21 to 150 days. The enrichment procedure afforded consistent identification of over 2000 parasitic peptides from the mixed-organism sample, representing 366 *T. gondii* proteins at 28, 90, and 120 day timepoints. Deep sequencing of transcripts expressed during these three timepoints revealed that a subpopulation of genes that are transcriptionally expressed at a high level. Approximately one-third of these transcripts are more enriched during bradyzoite conditions compared to tachyzoites and approximately half are expressed at similar levels during each phase. The *T. gondii* transcript which increased the most over the course of chronic infection, sporoAMA1, shows stage specific isoform expression of the gene.

**Conclusions:**

We have expanded the transcriptional profile of in vivo bradyzoites to 120 days post-infection and provided the first in vivo proteomic profile of *T. gondii* bradyzoites. The RNA sequencing depth of in vivo bradyzoite *T. gondii* was over 250-fold greater than previous reports and allowed us to identify low level transcripts and a novel bradyzoite-specific isoform of sporoAMA1.

## Background

The protozoan parasite *Toxoplasma gondii* is one of the most successful eukaryotic pathogens, infecting approximately a quarter of the world’s population [1]. One of the drivers of its success as a pathogen is the ability to develop a chronic infection in the brain of any warm-blooded host. Within the brain, the parasite undergoes a transformation from the fast-growing tachyzoite form to the slow-growing bradyzoite form [2]. Bradyzoites remain shielded from the host immune system by changing surface protein expression and developing a cyst wall [2, 3]. *T. gondii* cysts cannot be cleared from the host and current therapeutics are ineffective against chronic infection. This persistence becomes particularly problematic if the host becomes immunocompromised. As the immune pressure wanes, bradyzoites transition back into tachyzoites and start to rapidly replicate, which can lead to encephalitis if left untreated [4]. Despite its clinical importance, the biology of bradyzoites and the molecular components of the cyst structure are not well understood.

*T. gondii* undergoes a major transformational change during the switch from a tachyzoite to a bradyzoite. The transition has been characterized by both microarray analysis of cysts 21 days post-infection (DPI) [5] and deep sequencing of cysts 28 DPI [6], which identified ~ 500–800 genes changing between the different stages in vivo. This data has provided a good snapshot of cysts during early chronic infection; however, bradyzoites have a dynamic growth pattern during chronic infection. Parasite growth measured by ICM3 protein abundance showed a cyclic growth pattern of bradyzoites out to 8 weeks post-infection [7]. These results suggest that cysts are not static but are continually changing and responding to the environment.

The documented changes in cysts throughout chronic infection has led us to question whether the global transcriptional profile of bradyzoites changes over the course of chronic infection. For this study, we isolated *T. gondii* cysts from the infected brain tissue of mice 21, 28, 90, and 120 DPI using dextran centrifugation. This purification method resulted in enough parasite material to analyze both the global RNA and protein profile of in vivo bradyzoites. While the proteins present on the cyst wall have been recently analyzed [8], this current study is the first proteomic analysis of *T. gondii* bradyzoite parasites and includes the transcription profile of *T. gondii* bradyzoites throughout the time course of chronic infection.

## Results

### Enrichment of bradyzoite cysts decreases host protein interference

**Fig. 1:**
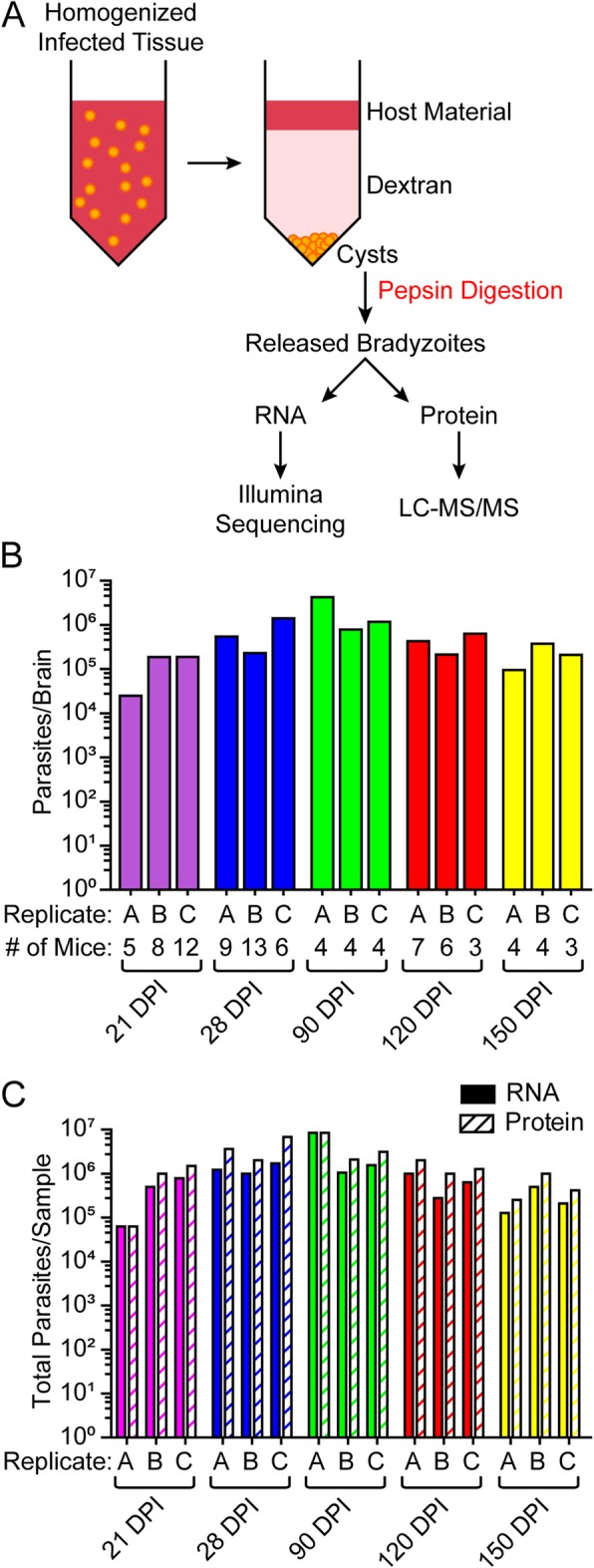
*T. gondii* bradyzoites are enriched by dextran. **a** Schematic of the bradyzoite enrichment workflow to enrich for parasites for RNA and protein sequencing. **b** The number of parasites per brain of each replicate labeled (**a**, **b** and **c**). Samples consisted of bradyzoites collected from a pool of homogenized brains and the number (#) of mice in each pool is shown. **c** Number parasites used for either RNA (solid colored bars) or protein (striped bars) analysis

*T. gondii* bradyzoite cysts were enriched from homogenized brain of host mice using dextran density centrifugation [9] for in vivo RNA-seq and proteomic analyses (Fig. 1a). Each replicate was a pool of several mouse brains (ranging from 3 mice to 13 mice per group, Fig. 1b). Bradyzoites were released from cysts by pepsin digestion. Approximately two-thirds of the parasites from each replicate were processed for protein analysis and one-third was processed for RNA (Fig. 1c). The highest parasite burden was at 28 and 90 DPI, and the lowest burden at the earliest (21 DPI) and latest (150 DPI) timepoints (Fig. 1b). As the chronic infection progresses, survival decreases [10], which limited the number of mice harvested at the latest timepoints. With few mice and lower parasites per brain, the 150 DPI timepoint resulted in the fewest total parasites collected (Fig. 1c).

**Fig. 2:**
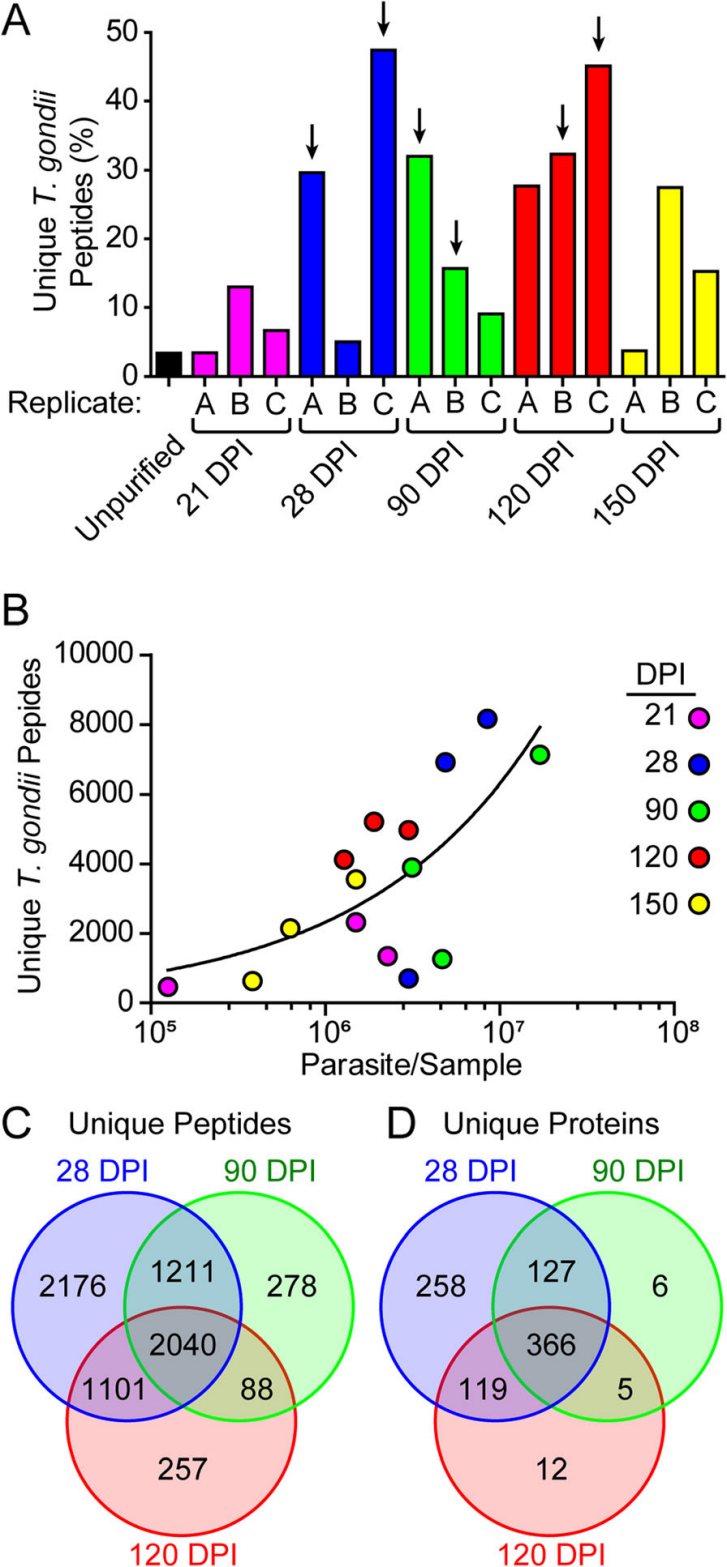
Parasite number affects *T. gondii* peptide abundance. **a** Percentage of unique *T. gondii* peptides from the total number of unique peptides identified in each sample. Arrows represent samples used for RNA-seq analyses. **b** Correlation of the number of parasites present in each sample compared against the number of unique *T. gondii* peptides in that sample. The line represents the log-log nonlinear regression curve (*r*^*2*^ = 0.50). The number of unique peptides (**c**) and proteins (**d**) identified in two samples at each timepoint (arrows from panel **a**). DPI = days post-infection

Protein samples were analyzed by bottom-up LC-MS/MS to identify parasitic and host proteins. The total number of unique peptides ranged from 11,548 to 24,833 (Additional file 1). Dextran purification of the infected brains increased the number of peptides that mapped to the *T. gondii* genome for many of the samples. Without dextran purification, protein from infected brain tissue mapped 3.4% of the peptides to *T. gondii* (black bar, Fig. 2a), whereas many purified samples mapped more than 25% of the peptides to *T. gondii*. Notably, the 21 DPI samples were near the same *T. gondii* mapping percentage as unpurified tissue, possibly because the bradyzoite cysts were not mature enough to sediment under the dextran. The number of unique *T. gondii* peptides ranged from 462 at 21 DPI, to 8163 at 28 DPI, and correlated with the number of bradyzoites in the sample (Fig. 2b). These peptides mapped to 1683 unique *T. gondii* proteins (Additional file 2). We chose two samples in each of the middle timepoints (28, 90 and 120 DPI) with the highest *T. gondii* peptide count and enrichment for further analysis (Fig. 2a arrows). From these samples, we required peptides be identified in both replicates of each timepoint. This resulted in 6528 peptides identified at 28 DPI, 3617 at 90 DPI and 3486 at 120 DPI, with 2040 peptides common between all three timepoints (Fig. 2c). The identified peptides represent 870 proteins at 28 DPI, 504 proteins at 90 DPI, and 502 at 120 DPI for a total of 893 unique proteins with 366 common between all timepoints (Fig. 2d, Additional file 3).

### StringTie program predicts novel T. gondii bradyzoite transcripts

RNA from the six samples with the highest percentage of *T. gondii* peptides (Fig. 2a arrows) were processed for Illumina sequencing. Samples were multiplexed and sequencing acquired over 265 million total reads, averaging 44 million reads per sample (Table 1). These samples were highly enriched for *T. gondii*, as 52–94% of the reads aligned to the *T. gondii* genome (21–41 million *T. gondii* reads per sample). This coverage is comparable to that of tissue culture tachyzoites, which ranged from 45 to 83% reads aligning to *T. gondii* (approximately 20 million total reads). This coverage is 265-fold greater than the number of reads acquired from infected whole brain tissue [6], which mapped only 0.1% of reads to *T. gondii*.

**Table 1:** RNA-seq reads per sample. The number of total reads sequenced from each sample and the number of which mapped to the *T. gondii* genome

Sample Type	DPI	Replicate ID	Input Reads	Toxo Reads	% Toxo	Reference
Tachyzoites	0	1	25837455	11650902	45.09	This Study
		2	18310484	15269361	83.39	This Study
Whole Brain	10	1	102581171	116278	0.11	[6]
		2	125546828	97611	0.08	[6]
		3	81630704	51309	0.06	[6]
Whole Brain	28	1	103765423	167181	0.16	[6]
		2	113094439	137975	0.12	[6]
		3	114177191	162423	0.14	[6]
Bradyzoites	28	A	40656512	21075587	51.84	This Study
		C	39129732	36606828	93.55	This Study
Bradyzoites	90	A	37700412	35412305	93.93	This Study
		B	41174415	25965460	63.06	This Study
Bradyzoites	120	B	46857160	40734629	86.93	This Study
		C	45074363	34443785	76.42	This Study

**Fig. 3:**
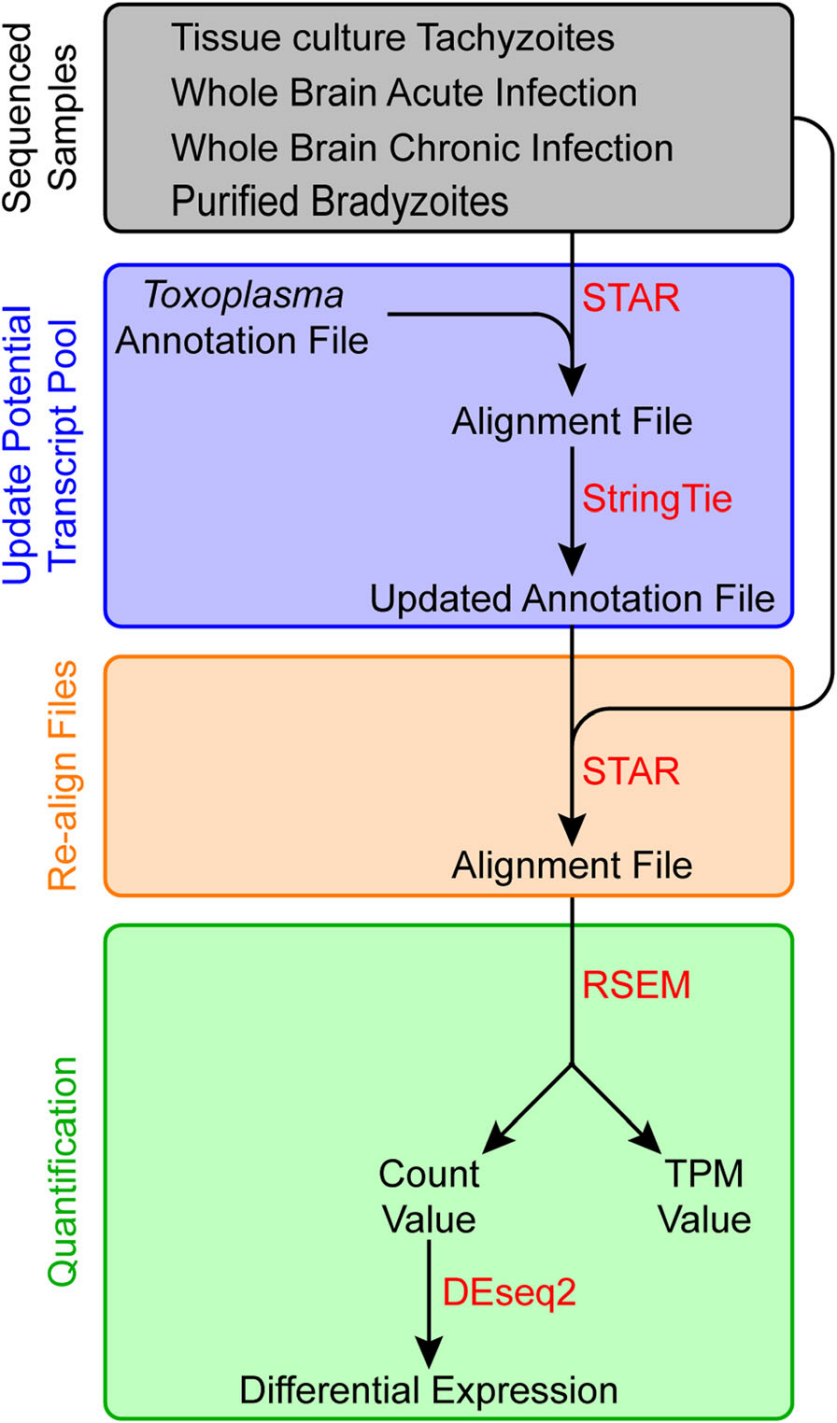
Schematic representing the RNA-seq workflow. Samples include tissue culture tachyzoites (*n* = 2), whole brain tissue from 10 DPI acute (*n* = 3) and 28 DPI chronically (*n* = 3) infected mice, and purified bradyzoites from 28 (*n* = 2), 90 (*n* = 2), and 120 (*n* = 2) DPI

To obtain higher transcript resolution for the bradyzoite datasets, we used the StringTie program [11] to predict novel transcripts (Fig. 3). The alignment files of all datasets (Table 1) were used to update the original *T. gondii* annotation. This update changed the number of predicted genes from 8920 to 8805 and the number of predicted transcripts from 8920 to 10,668. This updated annotation was used to remap the reads. Subsequently, the gene (Additional file 4) and transcript (Additional file 5) abundance was quantified using RSEM and DESeq2 [12, 13].

**Fig. 4:**
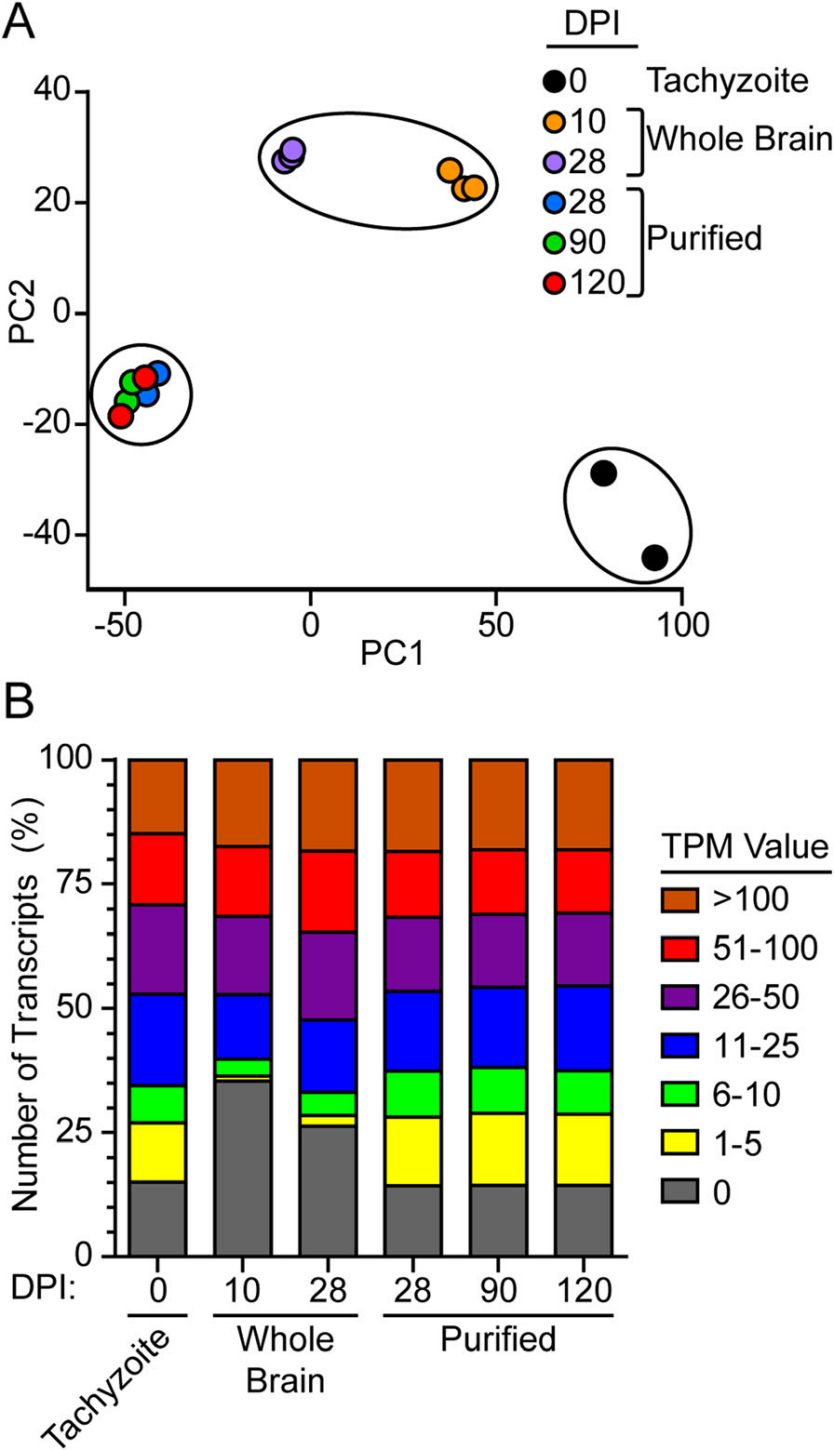
RNA-seq samples group with experimental design and with infection state. **a** PCA plot from normalized values calculated by DESeq2. Symbol colors represent: tissue culture tachyzoites (black); whole brain acute infection (orange); whole brain chronic infection (purple); purified at 28 days (blue); purified at 90 days (green); purified at 120 days (red). **b** Percent of transcripts with TPM values within each range: 0 (grey), 1–5 (yellow), 6–10 (green), 11–25 (blue), 26–50 (purple), 51–100 (red), and > 100 (brown)

The principal component analysis (PCA) calculated from the normalized DESeq2 values shows distinct clustering of samples by their experimental groups (Fig. 4a). Tissue culture tachyzoites group on the far right of the X-axis, while the purified bradyzoites group on the left. Whole brain tissue lays in the middle, with acutely infected samples skewed towards tachyzoites and chronically infected samples skewed toward the bradyzoites. All timepoints show a similar number of highly expressed transcripts with a normalized expression value (transcripts per million; TPM) > 50, as well as moderately expressed (TPM 11–50) transcripts. The overall number of low expression transcripts (TPM < 10) are only similar between tachyzoites and purified bradyzoites (Fig. 4b). The lower read depth of *T. gondii* reads in the unpurified brain samples likely contributed to the increase in undetected transcripts and thus a shift away from the purified 28-day bradyzoite samples on the PCA plot.

### Most identified proteins had high gene expression

**Fig. 5:**
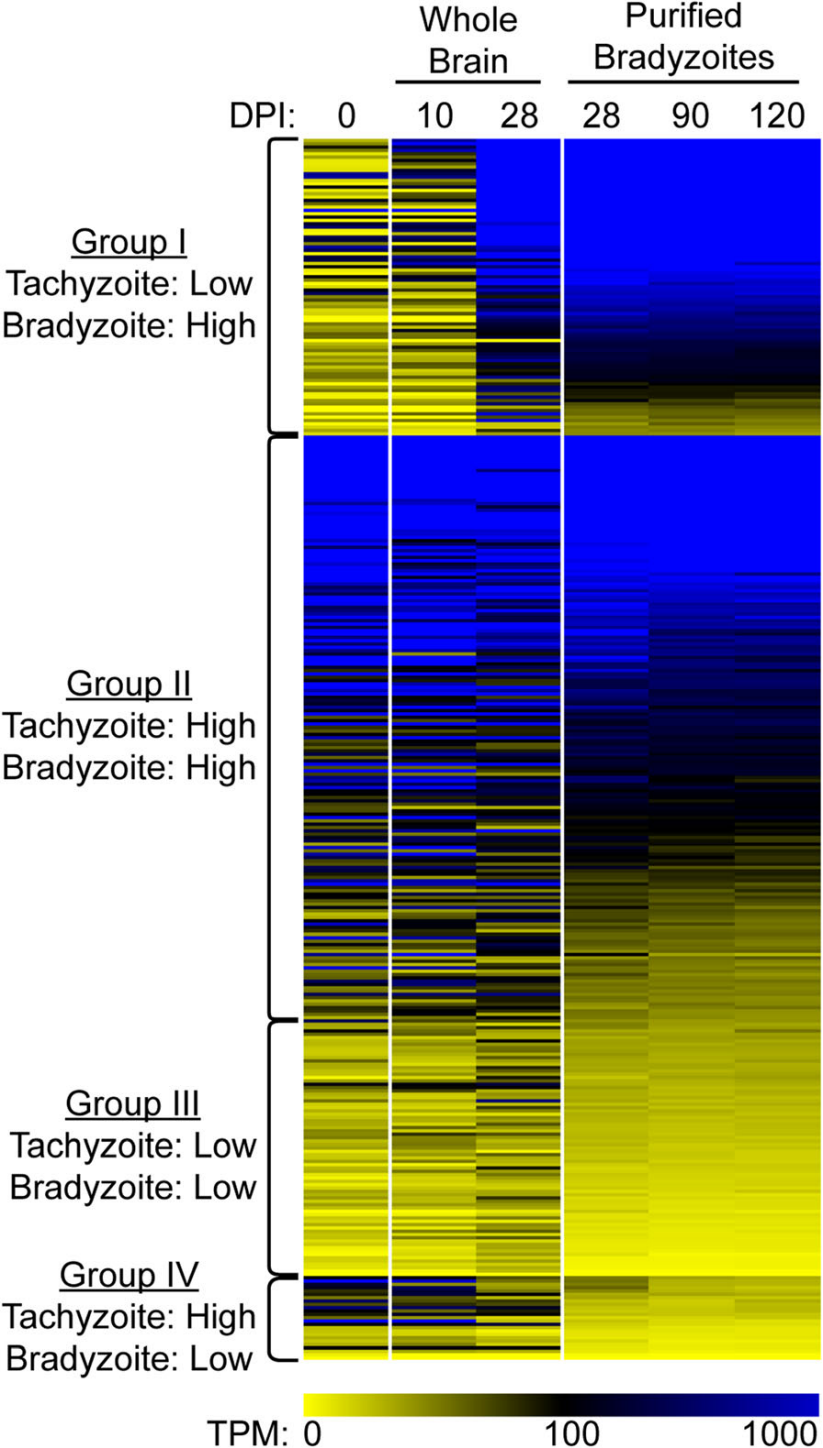
Most in vivo bradyzoite proteins have high gene expression. TPM values for the genes encoding the proteins identified in all six analyzed samples. Each row represents the gene for the protein and columns represent the sample type: tissue culture tachyzoites (0); whole brain acute infection (10); whole brain chronic infection (28); purified at 28 days (28); purified at 90 days (90); purified at 120 days (120). The TPM value is the average between the replicates of each group (*n* = 2–3). High expression is defined as an average TPM > 100 between samples under tachyzoite (0 and 10 DPI) or bradyzoite (28–120 DPI) conditions

We next analyzed normalized expression calculated from RSEM (TPM values). The ‘gene abundance’ output of RSEM is aggregated from all transcripts that map to each annotated gene. We focused on the 366 proteins identified by mass spectrometry that were common between all timepoints (Additional file 3). Of the 366 proteins, 266 were highly expressed in the purified and whole brain chronic infection samples with an average TPM > 100 (Fig. 5). Of these, 89 genes were > 2-fold higher during chronic infection relative to acute infection (Fig. 5, Group I, the average of all chronic TPM values relative to tachyzoite and acute whole brain sample). The genes in this subset include known bradyzoite markers such as BAG1, ENO1, and LDH2. Gene ontology (GO) enrichment analysis shows that DNA interaction and chromatin assembly GO terms are common in this group (Additional file 6), suggesting that bradyzoites require structural changes in its chromatin for bradyzoite gene expression. Also, the 200 highest expressed genes in the enriched bradyzoites had enriched GO terms related to translation and peptide synthesis (Additional file 6). Approximately half of the genes are highly expressed during both tachyzoite and bradyzoite stages (Fig. 5, Group II, > 100 average TPM value and < 2-fold difference between stages). These highly expressed genes include many housekeeping genes such as genes for tubulin, actin, and GAPDH as well as several metabolism related GO terms enriched among this group (Additional file 6). Interestingly, 100 proteins that were identified in all 6 bradyzoite samples had low gene expression (Fig. 5, Group III, < 100 TPM), with 25 of these genes are higher during acute infection (Fig. 5, Group IV). GO enrichment analysis of groups III or IV showed no GO terms significantly enriched.

### Transcriptomic analysis of purified bradyzoites results in a higher number of differentially expressed genes

**Fig. 6:**
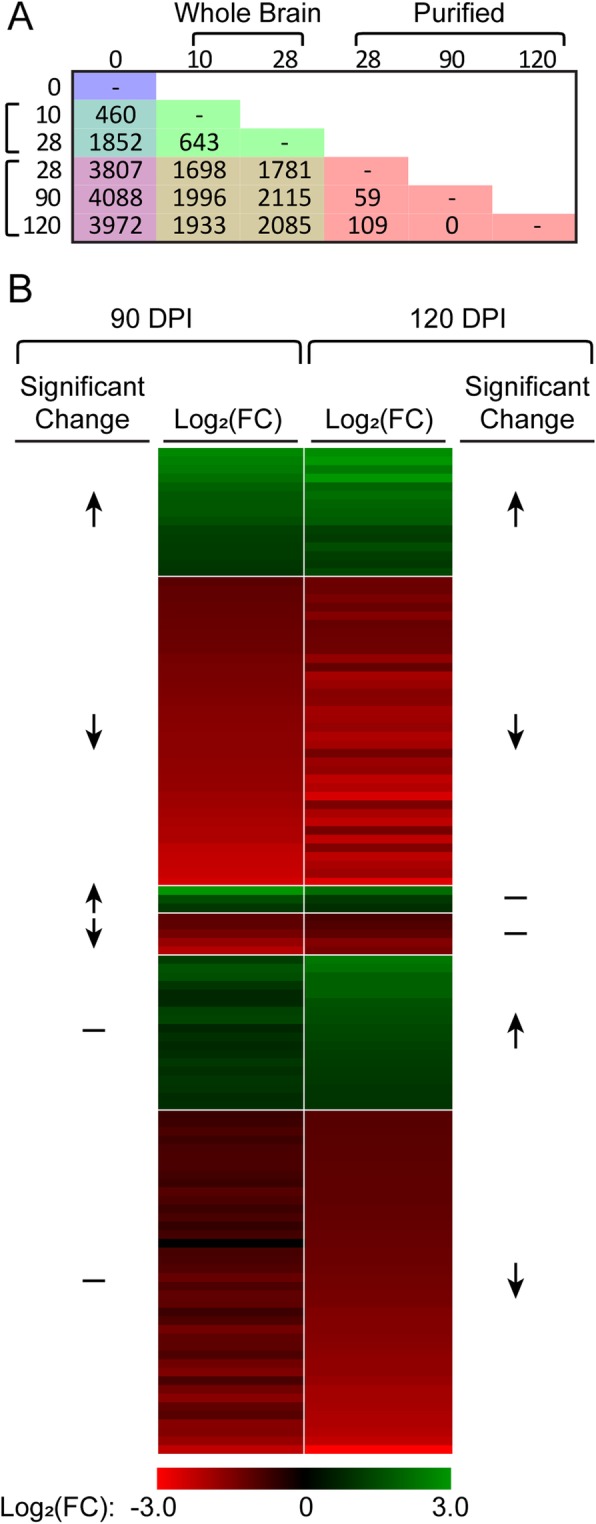
Differentially expressed transcripts during late-chronic infection. **a** The number of differentially expressed transcripts (> 2-fold difference and q-value < 0.05) among all conditions. **b** Differentially expressed transcripts during late-chronic infection. Log_2_ fold change values at 90 days post-infection (DPI) and 120 DPI relative to 28 DPI. Upward arrows indicate transcripts are significantly increasing at that timepoint, downward arrows indicate transcripts are significantly decreasing at that timepoint, and a dash indicates no significant change at that timepoint

As described above, we used the StringTie program to predict novel transcripts (Fig. 3). Using this updated annotation, we remapped the sequencing reads from our previous acute and chronic infection whole brain datasets to analyze differential expression with DESeq2. Using the new annotation, we identified 643 transcripts changing in abundance between acute and chronic whole brain tissues using a q-value (false discovery rate) threshold < 0.05 and 2-fold cut-off (Fig. 6a). This number compares to the 547 genes previously identified [6]. As mentioned, the dextran purification allowed greater depth of *T. gondii* sequencing. A direct comparison of *T. gondii* genes from whole brain tissue at 28 DPI with the purified bradyzoites from either the 28, 90 or 120 DPI resulted in approximately 2000 differentially transcribed genes (Fig. 6a, tan colored boxes). Comparing all three purified brain bradyzoite timepoints to tissue culture tachyzoites showed approximately 4000 of the transcripts changed during chronic infection (Fig. 6a, purple colored boxes).

### T. Gondii transcription remains static after 3 months post-infection

Comparing purified bradyzoites throughout chronic infection, 59 transcripts (from 50 genes) change at 90 DPI and 109 transcripts (from 93 genes) change by 120 DPI relative to 28 days (Fig. 6a, Additional files 7 and 8). No transcripts change significantly between 90 and 120 DPI. Of the differentially expressed transcripts, 51 transcripts were differentially expressed at both 90 and 120 DPI: 15 increasing in abundance and 36 decreasing (Fig. 6b). Only 8 transcripts change specifically at 90 days, whereas 58 transcripts are specific for 120 days: 18 increasing and 40 decreasing in expression. 33 of the differentially expressed transcripts have an average TPM value < 10 among all purified bradyzoite timepoints. This group of 33 included the transcript for SAG1 which had an average TPM value of 16 at 28 days, decreasing 3.5-fold and 4.8-fold at 90 and 120 DPI, respectively. This low level of bradyzoite expression is compared to the average TPM values of 8000 and 13,000 for tachyzoites and acute whole brain tissue respectively. Enriched GO terms among the genes that are reduced in expression during late-chronic infection relate to protein phosphorylation and protein modification. Specifically at 120 DPI, GO terms related to polysaccharide processes were enriched, suggesting much of the developmental changes are complete by 28 DPI.

**Fig. 7:**
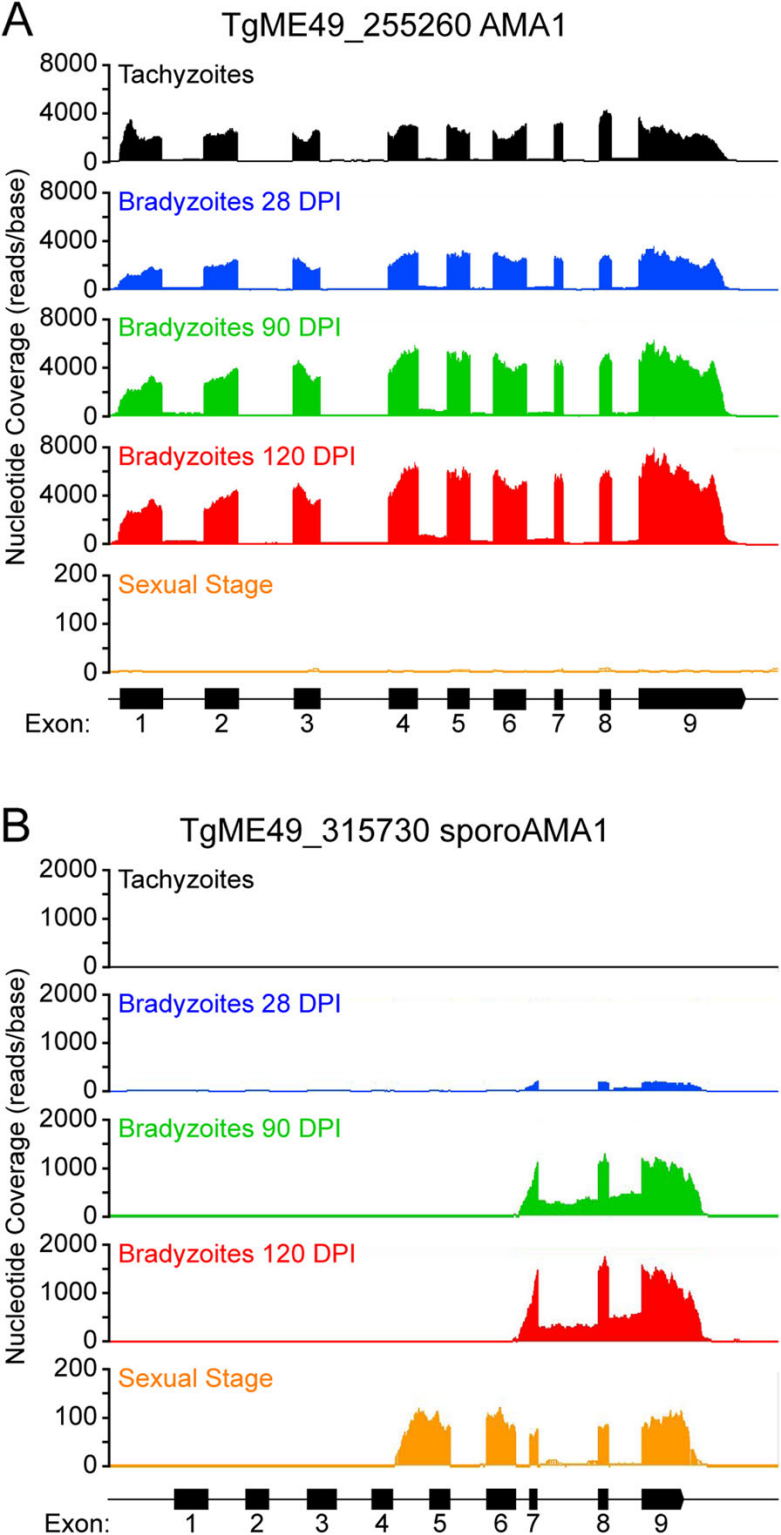
spoAMA1 has stage specific transcripts. Sequencing coverage from a representative sample from each group: Tachyzoite (black), purified bradyzoites at 28 DPI (blue), purified bradyzoites at 90 DPI (green), purified bradyzoites at 120 DPI (red). Sequencing coverage for *T. gondii* during infection in cat intestines is shown in orange. The X-axis represents the genomic region for AMA1 (**a**) and sporoAMA1 (**b**). The Y-axis represents the total read count at each nucleotide position. Predicted exons for the gene are represented by black rectangles under each panel

One of the transcripts which had one of the largest changes during chronic infection was for the gene sporoAMA1 (transcript MSTRG.4390.2, ToxoDB ID: TGME49_315730). SporoAMA1 was identified as a sporozoite specific paralog of the AMA1 protein, which is an essential component of the moving junction during invasion of the host cell [14, 15]. While most other transcripts map to the entirety of the predicted full gene, such as conventional AMA1 (TgME49_255260, Fig. 7a), reads to sporoAMA1 mapped to only the 3′ region of the gene and almost no reads mapped to the 5′ region (Fig. 7b). Coverage for all bradyzoite timepoints starts in the predicted intron region between exons 6 and 7. Compared to 28 DPI, both 90 and 120 DPI had an approximately 7.0-fold increase in transcript abundance. RNA-seq coverage from *T. gondii* 7 DPI in the intestines of cats [16] shows a similar coverage profile of sporoAMA1, except coverage starts at the predicted intron between exons 4 and 5. Both of these isoforms for sporoAMA1 are shortened from that of sporulated oocysts [17], which include all 9 exons (Additional file 9, Figure S1A). This coverage suggests that *T. gondii* has stage alternative isoforms of sporoAMA1 that are expressed only during late-chronic infection or the sexual stages in cats.

## Discussion

Although the host is largely asymptomatic during chronic *T. gondii* infection, bradyzoites are actively replicating within cysts [7]. Transcriptional activity of parasites during early chronic infection is high; however, the abundance of host material has hindered global analyses of *T. gondii* proteins and late-chronic transcripts. Our ability to collect tissue cysts and separate them from host material has allowed us the resolution to analyze low abundant *T. gondii* transcripts and to reproducibly identify many *T. gondii* peptides.

Dextran enrichment greatly increased the relative *T. gondii* peptide abundance. Unpurified infected brain found 3.4% of total unique peptides mapping to *T. gondii,* which increased to 30% for many of the enriched samples (Fig. 2a). Although some samples yielded fewer *T. gondii* peptides, comparing the most consistent samples between timepoints identified 2040 unique peptides in all samples, mapping to 366 different proteins (Fig. 2). While pepsin digestion removed host material and release bradyzoites from the cysts, many cyst wall proteins were likely lost due to the procedure. Despite the pepsin treatment, some known cyst wall markers, such as CST1 and GRA2, were seen [18, 19]. Also due to the enrichment procedure, we were unlikely to identify host proteins that were associated with the parasites or the cyst wall. It is interesting to note that two host transporter proteins associated with antigen processing (TAP1 and TAP2) were identified in every sample by MS/MS. Transcripts for these proteins are highly expressed in the brain during chronic infection [10], suggesting the TAP proteins may not be interacting directly with the cyst. In addition, 31 host proteins identified in our datasets were also identified from in vitro *T. gondii* cyst wall fractions (Additional file 2, right column) [8], suggesting there may be an association with the cyst wall among these proteins.

For many of the identified *T. gondii* proteins, the RNA expression levels were high during chronic infection (Fig. 5). These genes include the bradyzoite specific markers BAG1 and LDH2 [20, 21], as well as constitutively expressed genes such as Act1 and TubA1. Interestingly, 100 of the 366 proteins had low level gene expression during chronic infection. These results likely indicate either short-lived transcripts or long-lived proteins. Alternatively, as bradyzoites within tissue cysts are heterogeneously replicating in vivo [7], some proteins may be produced by a subset of metabolically active parasites or those undergoing reactivation into the tachyzoite state.

Cyst isolation has allowed us to identify transcripts from in vivo parasites at a depth similar to parasites in vitro. This depth allowed examination of alternative transcripts produced by bradyzoites from early to late chronic infection. Comparing timepoints with two biological replicates from each, over 100 transcripts were differentially expressed from early to late chronic infection. No transcripts change between 90 and 120 DPI, suggesting that, at the global level, the parasites are at a steady state by 90 DPI. This result is consistent with many host transcripts decreasing expression from 28 to 90 DPI and remaining similar from 90 to 180 DPI [10].

One of the transcripts which had the largest change from early to late infection was for sporoAMA1. The StringTie program identified two transcripts for sporoAMA1 (MSTRG.4390.2 and TGME49_315730-t26_1). Both transcripts were significantly upregulated at 90 and 120 DPI from 28 DPI, and MSTRG.4390.2 had the largest increase at near 7-fold increased at both late-chronic timepoints compared to 28 DPI. SporoAMA1 is the sporozoite specific paralog of the AMA1 protein and a major component of the moving junction during entry into the host cell [14, 15]. The N-terminal region of sporoAMA1 includes a binding domain to the *T. gondii* protein RON2 [14, 22] to help form the moving junction complex. The annotated gene for sporoAMA1 includes 9 exons (transcript TGME49_315730-t26_1); however, the bradyzoite reads do not align until exon 7 (Fig. 7b). In comparison, RNA-seq data from *T. gondii* infected cat intestines showed that the reads to sporoAMA1 do not align until after exon 4 (Fig. 7). Microarray evidence of this same region from sporulated oocysts [17], show reads aligning to all nine exons of sporoAMA1 (Additional file 9). This result suggests that sporoAMA1 has stage specific isoforms, with the expression of the shortest isoform starting in late chronic infection and longer isoforms expressed during different stages of the cat cycle.

### Future directions

The functions of the shortened sporoAMA1 isoforms are unknown. For each of these transcripts, an in frame start codon is present 150–300 base pairs after the reads start aligning to the genome, highlighting that these shortened transcripts may be translated. It is unlikely that either of these two short forms plays a role in invasion as the RON2 binding region is not present in either isoform (Additional file 9, Figure S1B). In support of a non-invasion function for sporoAMA1, there are almost no transcripts sporoRON2 (TgME49_265120) transcripts expressed either in bradyzoite cysts or sexual stages in the cat intestine. The shortest isoform would produce an 8 kDa protein during late chronic infection that contains the cytoplasmic region and the conserved serines/threonines. The sequential phosphorylation of these residues is essential for *Plasmodium falciparum* to efficiently invade red blood cells [23]. Thus the 8 kDa bradyzoite isoform may be phosphorylated and playing a regulatory role. However, no sporoAMA1 peptides were found in any of the six chronic infection cyst samples generated in this study. While this result could indicate that the short 8 kDa isoform is unstable or degraded in the sample preparation process, it could also indicate that the approximately 400 bp RNA molecule is a long non-coding RNA. Perhaps this RNA functions similar to the Herpes encoded Latency Associated Transcripts (LATs), which are essential to maintain viral latency and reactivation [24]. The generation of an antibody to this shared cytoplasmic domain will help determine if the short isoforms are translated as well as their potential function.

Further future studies are also needed to determine the host interactions with the bradyzoite cyst during long-term chronic infection. Upwards of 90% of the RNA-seq reads obtained in this study aligned to the *T. gondii* genome, whereas the 75% of the peptides aligned to the mouse. This difference is likely due to the instability of the host mRNA and the stability of the host proteins during sample processing. The enrichment of certain host proteins may indicate their stable interaction with the cysts. We have not yet examined if any identified host proteins are directly associated with the parasites or the cyst wall. The membrane associated host transporters TAP1 and TAP2 were identified in each sample by MS/MS. This result may be a consequence of their high expression in the brain during infection [10], or it may indicate a direct interaction with the cyst. *T. gondii* interaction with TAP1 and TAP2 could block antigen processing to help hide the cyst during chronic infection or it might assist in nutrient acquisition across the cyst wall. Our dataset had 31 host proteins (Additional file 2, right column) that were also identified in in vitro *T. gondii* cyst wall fractions [8], suggesting that these host proteins may be an association with the cyst wall. Examination of these host proteins potentially interacting with cysts will be an important area of future research as it may indicate how cysts can reside in their warm-blooded host for so long.

## Conclusions

The isolation of in vivo bradyzoites has allowed us to look deeply at both the transcript and protein content of the parasites. This depth has allowed us to identify novel bradyzoite specific isoforms, one of which is sporoAMA1. Over the course of chronic infection, we have found that the global transcriptional profile remains constant after the first month of chronic infection. This result is similar to the protein profile, in which most of the proteins identified are found in each timepoint. This dataset is the first global protein profile of in vivo bradyzoites. Together with the RNA-seq data, they provide a better understanding of the transcriptional and translational activity of *T. gondii* throughout infection.

## Methods

### Mouse infection with T. gondii

The ME49 strain of *T. gondii* was cultured as tachyzoites in human foreskin fibroblast (HFF) cells. Six to ten week-old CBA/J (JAX) were infected intraperitoneally with 1 × 10^4^ tachyzoites to establish chronic toxoplasmosis.

### Collection of tissue bradyzoites

At their respective timepoint, the mice were euthanized by carbon dioxide inhalation in a manner approved by the institutional animal ethics committee, and the whole brains of infected CBA/J mice at 21, 28, 90, 120, and 150 days post-infection were harvested and homogenized in phosphate buffered saline (PBS) using mortar and pestle. Several brain tissues were homogenized together as a single replicate to obtain enough parasite material. Homogenized tissue was serially passed through increasing gauge needles (18, 20, 22 respectively 5x each) and filtered using 150 μm filter. The volume of the homogenate was increased to 40 mL using PBS and centrifuged for 10 min at 2000 x g. Pellet was resuspended in 3 mL of 20% Dextran (150,000 average molecular weight; Alfa Aesar) per brain and separated into tubes so that 3 or fewer brains were in each. Samples were centrifuged for 10 min at 400 x g to pellet the cysts. Host material and dextran were removed, and the cyst pellet was resuspended with 5 μg/mL pepsin in a 1% NaCl solution at pH 2.1 to release the bradyzoites from the cyst (1 mL per brain). Cysts were digested for 5 min at 37 °C, then neutralized with a 1x volume of 1% Na_2_CO_3_. Bradyzoites were centrifuged for 10 min at 250 x g and resuspended in PBS then counted using hemocytometer.

### Protein LC-MS/MS

Bradyzoites were preserved in 6 M GuHCl prior to processing. Protein was isolated by boiling at 100 °C for 5 min before precipitating in 90% methanol. Protein was pelleted by centrifugation at 10,000 x g for 5 min, the supernatant was discarded, and the pellet was resuspended in lysis buffer (8 M urea, 100 mM tris pH 8, 40 mM 2-chloroacetamide, 10 mM 2-carboxyethyl phosphine). Samples were incubated for 10 min at RT before diluting to < 2 M urea with 50 mM tris pH 8. Trypsin (Promega, Madison, WI) was added at a mass ratio of 1:100::trypsin:protein and the sample was incubated overnight at RT with gentle rocking. A second bolus of trypsin (1:200::trypsin:protein) was added the next morning and incubated for an additional hour before desalting with reversed phase cartridges (StrataX, Phenomenex, Torrence, CA). Peptide yield was calculated by bicinchoninic acid (BCA) assay (Thermo Peirce, Rockford, IL). Two microgram of each sample was analyzed by LC-MS/MS on a Q-IT-OT Fusion Lumos mass spectrometer (Thermo Fisher, San Jose, CA) equipped with a 75/360 μm inner/outer diameter fused silica capillary column with a laser pulled electrospray tip packed with BEH C18 (130 Å pore, 1.7 μm particle size, 35 cm, Waters Corp, Milford, MA). Eluting peptides entered into the mass spectrometer following positive mode electrospray ionization. MS1 survey scans were performed in the orbitrap (240 K resolution, AGC target 1e6, 100 ms maximum injection time). MS2 analysis of HCD generated (25% NCE) product ions were performed in the ion trap (Rapid resolution, AGC target 4e4, 18 ms maximum injection time). Monoisotopic precursor selection and dynamic exclusion (60 s) were enabled. Raw data were searched against a concatenated database of mouse and *T. gondii* proteins protein sequences through the MaxQuant computational platform (version 1.6.0.13) operating the Andromeda search algorithm [25]. Precursor and fragment mass tolerances were set to 50 ppm and 0.5 Da, respectively. Carbamidomethylation of cysteine was imposed as a fixed modification and oxidation of methionine was allowed as a variable modification. Protein inference was performed from peptide spectrum matches (PSMs) based on the rules of parsimony [26]. Search results were filtered to a 1% false discovery rate at both the PSM and protein levels.

### Generation of RNA and RNA-seq

Trizol was added to bradyzoites for the preservation of RNA, and the RNA extracted using phenol/chloroform separation and isopropanol precipitation. Samples include brain enriched bradyzoites at 28 DPI (*n* = 2), 90 DPI (*n* = 2), and 120 DPI (*n* = 2). Ten nanogram of total RNA was processed using the SMART-Seq v4 Ultra Low Input RNA Kit (Takara), using 7 rounds of cDNA amplification. One nanogram of cDNA was indexed using the Nextera XT Index Kit (Illumina). RNA libraries were multiplexed and run across 2 lanes for paired end, 125 base pair sequencing by Illumina HiSeq 2500 at the University of Wisconsin Biotechnology Center.

Tachyzoite RNA was processed from the ME49 parasites grown in human foreskin fibroblasts (HFF) cells. Just before lysis of the parasite vacuole, infected HFF cells were scraped in trizol and RNA extracted as stated above. RNA libraries were prepared using a TruSeq RNA Library Prep kit v2 set A (Illumina) and sequenced using paired-end sequencing as stated above.

### Transcriptome assembly

The sequencing reads were processed to remove low quality reads (Trimmomatic, v0.39 [27]). The tachyzoite, purified bradyzoite, and whole brain acute and chronic [6] sequencing reads were aligned to the *T. gondii* ME49 genome (ToxoDB.org) using the Spliced Transcripts Alignment to a Reference program (STAR, v2.7.0f, [28]). The following STAR parameters were changed from the default: mismatch max = 2, intron min = 70, and intron max = 100,000. The STAR output bam file from each sample was fed into the StringTie program (v1.3.6, [11]), along with the current *T. gondii* annotation file (ToxoDB.org) and using a minimum transcript coverage value of 150. The sequencing reads were remapped using the new gtf file output from StringTie. Transcript abundance was estimated using RNA-seq by Expectation-Maximization (RSEM v1.2.31, [12]). Fold change was calculated using the geometric mean values between samples for transcripts with at least 10 RSEM gene counts in any one sample. The rlog transformed DESeq2 values were used for the PCA plot and PCA calculated using “plotPCA” function of the DESeq2 package. GO term enrichment was perfomed using ToxoDB. Terms were limited to biological processes with a *p*-value < 0.05 and false discovery rate < 0.05.

## Supplementary information

**Additional file 1: Table S1.** Unique peptides identified from bradyzoites. Data shows either *T. gondii* or mouse peptides identified in each sample with their spectral count value. The pooled sample represents peptides identified when protein from all samples is mixed before processing for LC-MS/MS.

**Additional file 2: Table S2.** Unique proteins to which the identified peptides map to. Data shows proteins that were mapped to either *T. gondii* or mouse in each sample with their total spectral count value.

**Additional file 3: Table S3.** Proteins common between all bradyzoite samples. Data shows each protein with the protein spectral count for the six samples chosen for analysis.

**Additional file 4: Table S4.** Normalized sequencing read values for the *T. gondii* isoforms. TPM values calculated by RSEM for individual isoforms after aligning reads to the transcripts identified by StringTie. TZ, tissue culture tachyzoites; WholeBrain, data from whole tissue sequencing from either acute or chronically infected brain tissue; BZ, enriched in vivo bradyzoites.

**Additional file 5: Table S5.** Normalized sequencing read values for the sum of all *T. gondii* gene isoforms. TPM values calculated by RSEM for each gene after aligning reads to the transcripts identified by StringTie. TZ, tissue culture tachyzoites; WholeBrain, data from whole tissue sequencing from either acute or chronically infected brain tissue; BZ, enriched in vivo bradyzoites.

**Additional file 6: Table S6.** Enriched GO terms. GO terms significantly enriched among the highest expressed genes, chronically differentially expressed genes, and the groups of differently expressed genes among the proteins identified.

**Additional file 7: Table S7.** Log2 values of the fold change for differentially expressed isoforms. Values were calculated with DESeq2. Values are only shown if there was > 2-fold change between samples with q-value < 0.05. Values below the threshold are represented by “-“.

**Additional file 8: Table S8.** DE genes in chronic infection. Log2 values of the fold change for differentially expressed genes. Values were calculated with DESeq2. Values are only shown if there was > 2-fold change between samples with q-value < 0.05. Values below the threshold are represented by “-“.

**Additional file 9: Figure S1.** Different isoforms of sporoAMA1 are expressed during chronic infection, during the cat intestinal stage, and during oocyst sporulation. (A) All exons of sporoAMA1 are expressed during oocyst sporulation. Sequencing coverage from a representative sample from each group viewed from the ToxoDB genome browser: purified bradyzoites at 120 DPI (red), cat stage (orange), and oocyst microarray data at 0, 4, and 10 days post-sporulation (Brown). The X-axis represents the genomic region for sporoAMA1. The Y-axis represents the total read count at each nucleotide position. Predicted exons for the gene are represented by black rectangles under each panel. (B) Protein domains for the sporoAMA1 isoforms. DI, Domain I highlighted in red; DII, Domain II in green; DIII, Domain III in yellow; TM, Transmembrane region in blue; CPD, cytoplasmic domain in orange. Red arrows represent phosphorylation sites and black arrow represents the RON2 binding site.

## Data Availability

All raw RNA sequencing data and differential expression values have been deposited in NCBI’s Gene Expression Omnibus (GEO) [29] and are accessible through GEO Series accession number GSE134099. RNA sequencing and protein sequencing data have been supplied for public availability to ToxoDB.org.

## Abbreviations

DPI: Days post-infection
GO: Gene ontology
HFF: Human foreskin fibroblast
LC-MS/MS: Liquid chromatography-tandem mass spectrometry
PBS: Phosphate buffered saline
PCA: Principal component analysis
RNA-seq: High-throughput RNA sequencing
STAR: Spliced Transcripts Alignment to a Reference program
TPM: Transcripts per million
