# Supplementary figures and images for: Proteomic and transcriptomic analyses of early and late-chronic Toxoplasma gondii infection shows novel and stage specific transcripts
Source: BMC Genomics. 2019 Nov 14;20:859. doi: 10.1186/s12864-019-6213-0 (PMC6857164; doi:10.1186/s12864-019-6213-0)

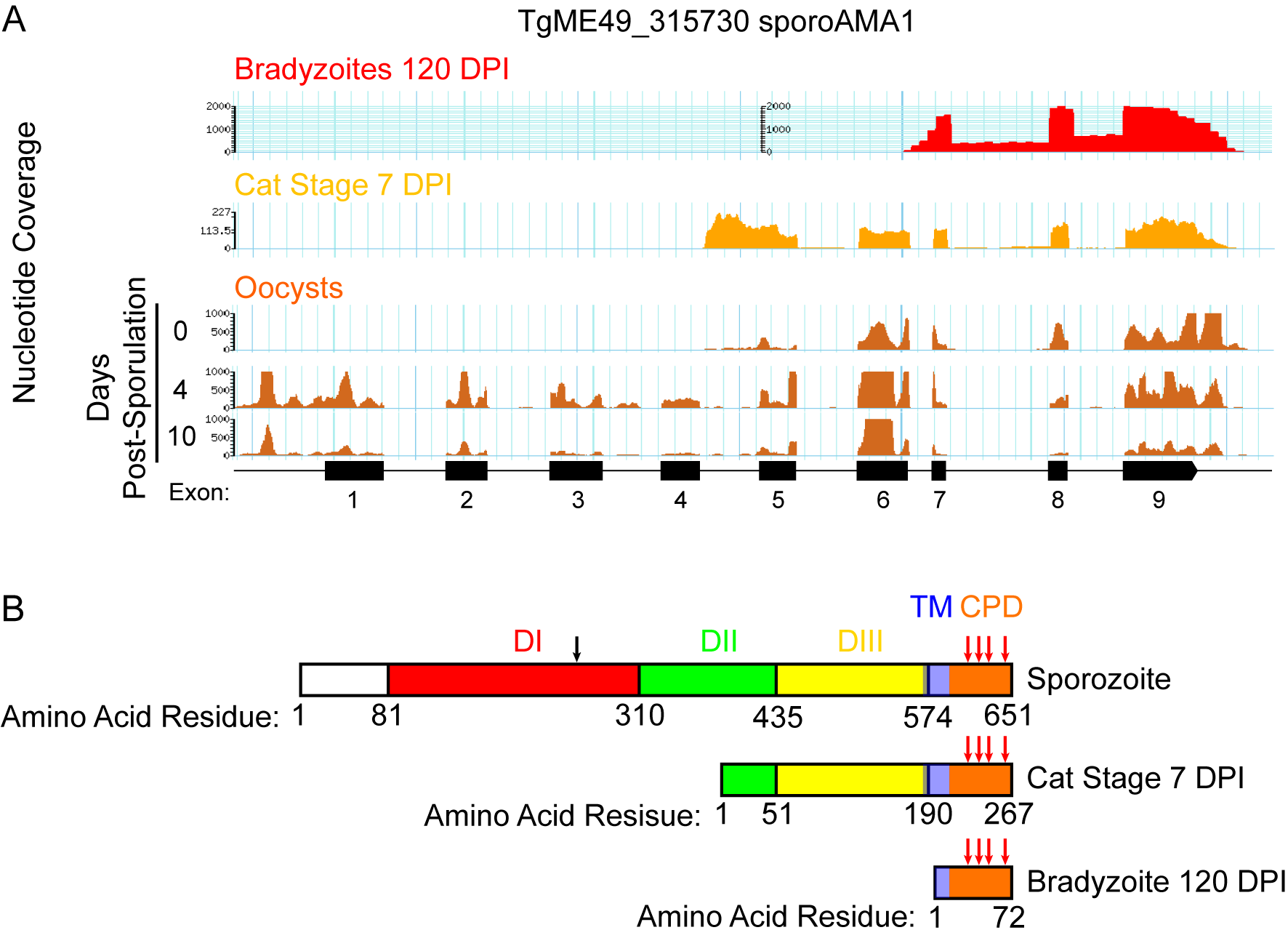

Supplement: Supplementary file 9 — Additional file 9: Figure S1. Different isoforms of sporoAMA1 are expressed during chronic infection, during the cat intestinal stage, and during oocyst sporulation. (A) All exons of sporoAMA1 are expressed during oocyst sporulation. Sequencing coverage from a representative sample from each group viewed from the ToxoDB genome browser: purified bradyzoites at 120 DPI (red), cat stage (orange), and oocyst microarray data at 0, 4, and 10 days post-sporulation (Brown). The X-axis represents the genomic region for sporoAMA1. The Y-axis represents the total read count at each nucleotide position. Predicted exons for the gene are represented by black rectangles under each panel. (B) Protein domains for the sporoAMA1 isoforms. DI, Domain I highlighted in red; DII, Domain II in green; DIII, Domain III in yellow; TM, Transmembrane region in blue; CPD, cytoplasmic domain in orange. Red arrows represent phosphorylation sites and black arrow represents the RON2 binding site. [file 12864_2019_6213_MOESM9_ESM.tif]
